# Adolescents’ and parents’ affect in relation to discrepant perceptions of parental warmth in daily life

**DOI:** 10.1111/jora.12879

**Published:** 2023-08-09

**Authors:** Loes H. C. Janssen, Carlie J. Sloan, Bart Verkuil, Lisanne A. E. M. Van Houtum, Mirjam. C. M. Wever, Gregory M. Fosco, Bernet M. Elzinga

**Affiliations:** 1 Department of Clinical Psychology, Leiden University, Leiden, the Netherlands; 2 Leiden Institute for Brain and Cognition (LIBC), Leiden, the Netherlands; 3 Human Development and Family Studies, The Pennsylvania State University, University Park, Pennsylvania, USA; 4 Edna Bennett Pierce Prevention Research Center, The Pennsylvania State University, University Park, Pennsylvania, USA

**Keywords:** daily diary, divergence, parental warmth

## Abstract

The current study aimed to evaluate how adolescents’ and parents’ perceptions of daily parenting—and their discrepancies—relate to daily parent and adolescent affect. Daily parental warmth and affect were assessed using electronic diaries in 150 American adolescent–parent dyads (61.3% females, *M*_age_ = 14.6, 83.3% White; 95.3% mothers, *M*_age_ = 43.4; 89.3% White) and in 80 Dutch adolescents with 79 mothers and 72 fathers (63.8% females, *M*_age_ = 15.9, 91.3% White; *M*_age_ = 49.0, 97.4% White). Results of preregistered models indicated that individuals’ affect may be more important for perceptions of parenting than discrepancies between parent–adolescent reports of parenting for affect, stressing the need to be aware of this influence of affect on parenting reports in clinical and research settings.

## INTRODUCTION

Adolescence represents a time when developing youth begin to gain independence outside of the family, negotiating new rules, freedoms, and relationships (Branje, 2018). During this period, changes to the parent–adolescent relationship are common and normative, often resulting in increased adolescent–parent conflict (Steinberg & Morris, 2001). Despite these increases in conflict, warm and supportive parenting remains important for adolescent well-being throughout adolescence. In general, adolescents from families characterized by higher levels of parental warmth are at lower risk for internalizing and externalizing problems (Pinquart, 2017; Rothenberg et al., 2020). Studies examining the daily dynamics of adolescents and their families, which have the power to elucidate within-person processes (i.e., individual changes over time), have converged on similar findings: on days when adolescents report more warmth or support from parents, adolescents also report higher positive and lower negative affect (Bai et al., 2017; Flook, 2011; Janssen, Verkuil, et al., 2021; Robles et al., 2016). However, exclusively relying on adolescent reports of parenting ignores the fact that family dynamics are the result of multiple family members’ perspectives, attitudes, and behaviors, as well as the interactions among them (Cox & Paley, 1997; Minuchin, 1985).

Recent multi-informant studies (e.g., using both adolescent and parent reports) have indicated that parents’ perception of their own parenting behavior can differ from adolescents’ perceptions (Brinberg et al., 2017; Hou et al., 2018, 2020; Korelitz & Garber, 2016), and have started to examine the impact that parent and adolescent perceptions of parenting behaviors, as well as differences between reports, have on adolescent affect. Despite the strong family systems theory emphasis on interrelatedness of family members and their behaviors (Cox & Paley, 1997; Minuchin, 1985), few studies have addressed how both parent and adolescent perceptions of parenting may contribute to fluctuations in daily mood, with most studies examining only adolescent reports. Additionally, a paucity of studies explore influences on parents’ mood in conjunction with adolescent mood, despite empirical evidence that parental affect and parenting are related (Rueger et al., 2011). In this paper, we aim to examine whether fluctuations in daily affect of both parents and adolescents are related to adolescents’ and parents’ perceptions of parenting and discrepancies between them. More insight into these family dynamic processes might ultimately help to inform interventions to promote positive family and individual well-being.

### Discrepancies and well-being of adolescents and parents

Previous research, using retrospective questionnaire data, has shown that discrepancies in parent–adolescent reports of parental warmth are associated with poorer adolescent well-being in general (e.g., Hou et al., 2020). The majority of studies indicate that this is specifically the case when adolescents perceive parenting more negatively than parents themselves. As individuals’ feelings and perceptions may vary considerably from day- to-day (e.g., Fosco et al., 2019) assessing these processes on the daily level is necessary. A recent study by Janssen, Verkuil, et al. (2021) focusing on these within-person fluctuations found that, in addition to individual perceptions of the adolescent, discrepancies in parent and adolescent reports of daily warmth (with adolescents reporting less parental warmth than their parents) was related to adolescents’ elevated negative affect and reduced positive affect on the same day. Overall, these results seem to suggest that discrepancies may be a marker for dysfunctional family dynamics that threaten adolescent well-being (De Los Reyes et al., 2019). However, it may also signify that the adolescent is gaining an individual identity and experiencing a normative decline in closeness to the family (Bowen, 1978; Grovetant & Cooper, 1985). In addition to the impact on adolescents’ well-being, it has been suggested that discrepancies may also undermine parents’ well-being (e.g., De Los Reyes, 2011), although this is rarely tested. According to family systems theories (Cox & Paley, 1997; Minuchin, 1985) and dynamic systems approaches (Granic et al., 2003), the parent–adolescent relationship can be seen as a dynamic system. A few studies have already highlighted that both adolescent and parent perceptions of the quality of their relationship in daily life are influential for the well-being of each dyad member (Fosco et al., 2021; LoBraico et al., 2020), but the extent to which discrepancies in parent–adolescent reports of parental warmth relate to parents’ well-being remains unknown. Therefore, the current study aims to add value by understanding the implications of parent–youth discrepancies for parent well-being in daily life. Instead of focusing on assessing positive and negative affect, which is a common practice, we aimed to focus on specific affect states: happiness, irritation, and sadness. Happiness is one of the most experienced affect states by adolescents (Bailen et al., 2019). Although irritation and sadness are less commonly experienced by adolescents (Bailen et al., 2019), these daily negative affect states can be important precursors for psychological problems such as internalizing problems (Maciejewski et al., 2017, 2019).

### Interrelatedness between adolescent and parent affect

Not only do adolescents’ and parents’ perspectives and behaviors interact, but their affective states can also influence each other (Dix, 1991; Rueger et al., 2011). The few studies that have examined the shorter-term interrelatedness of family members’ affect in daily life have found modest correlations between parents’ and adolescents’ affect on the daily level (Larson & Richards, 1994). A recent daily diary study indicated that happiness of adolescents’ and parents on the same day were related (Mercado et al., 2019). These associations can be due to interpersonal emotion dynamics (e.g., Butler, 2011), wherein affective states (e.g., happiness) in one person may elicit affect in the other person, but it may also be the case that both family members are involved in an event that elicits happiness in both (e.g., a nice family dinner). More research is necessary to examine whether the interrelatedness of parents’ and adolescents’ affect is associated with daily fluctuations in family contextual factors, such as parenting behaviors. By using intensive longitudinal methods, the current study aims to provide more insight into the dynamic processes around individual perceptions of parenting and discrepancies within a family and its relation to affect in daily life. Repeated measures allow individuals to be compared to their own averages across time, in order to assess the implications of having days with relatively more positive or negative perceptions of parental warmth compared to a usual day for that individual.

Additionally, our understanding of the relations between parenting and parent and adolescent affect so far is rather limited, because research is mainly based on reports about mothers. Although mothers and fathers might serve different and unique roles in parenting their adolescents (e.g., Lamb & Lewis, 2013), with mothers being more emotion-directed and supportive than fathers during adolescence (De Goede et al., 2009; Mastrotheodoros et al., 2018), parenting studies that include fathers remain scarce. Interestingly, the limited available data suggests that fathers’ affect may be more strongly associated with child affect than mothers’ affect in daily life, at least on average (Almeida et al., 1999; Larson & Richards, 1994). Regarding parenting, one recent study based on data that is also used in the current study found that discrepancies between adolescents’ and mothers’ reports of parental warmth were more consistently related to adolescent positive affect than discrepancies between adolescents and fathers (Janssen, Verkuil, et al., 2021). However, the association with mothers’ and fathers’ own affect was not yet investigated. Therefore, in addition to including both adolescents and parents, examination of possible parent gender differences in the implications of daily informant discrepancies is a key direction for research that we aim to assess.

Utilizing multiple informants and rigorous statistical methods fit for intensive longitudinal designs can move the field toward a more solid understanding of the interplay between daily adolescent and parent perceptions of the family and daily well-being. Recently, a hybrid (combined) statistical model, which enables including both the difference score and individual perceptions in one model, was proposed (Iida et al., 2018). This model combines advantages of the Actor Partner Interdependence Model (APIM; Kashy & Kenny, 1999) and Dyadic Score Model (DSM; Iida et al., 2018). Using the hybrid model, researchers are able to not only assess the extent to which a pair of exploratory variables (i.e., adolescent and parent perceptions of parental warmth) affect a pair of outcome variables (i.e., adolescent and parent affect), as with APIM, but also include a variable that characterizes a dyadic relationship, such as the discrepancy between adolescent and parent reports, as with DSM. The use of differences scores alone—that is, without taking into account each informant’s actual report of the construct, such as the degree of parental warmth—is insufficient to understand the impact of degree of divergence between two informant reports as the individual perceptions are ignored (see Laird & de Los Reyes, 2013). We therefore aim to use the hybrid model to assess how both the difference score and individual perceptions of daily parental warmth of parents and adolescents are related to adolescents’ and parents’ daily affect.

### The current study

The current study aims to investigate two research questions: (1) whether and how adolescents’ and parents’ perceptions of parental warmth, as well as discrepancies in adolescent-parent reports, are related to daily adolescent and parent affect, and (2) whether these associations differ between adolescent–mother and adolescent–father dyads. These questions are evaluated in two samples: one sample of 150 American parent–adolescent dyads (sample I—Family Life Optimizing Well-being (FLOW) study, *n* = 143 mother–adolescent and *n* = 7 father–adolescent dyads), and a second sample of 80 Dutch families in which in almost all cases both mothers and fathers completed the study (sample II—Relations and Emotions in Parent Adolescent Interaction Research [RE-PAIR], *n* = 79 adolescent–mother and *n* = 72 adolescent–father dyads). Capitalizing on a two-s ample design allows for replication of findings across samples with two different cultural contexts (i.e., testing research question 1 in both samples), as well as analysis of parental gender differences in effects which is only possible in sample II.

Both samples utilized a daily diary design to assess parenting that enabled us to assess how daily fluctuations in perceptions of parental warmth and parent–adolescent discrepancies are related to adolescent and parent affect and vice versa. Previous single time point studies have examined the implications of parenting and divergence in parent–youth reports at a between-family level (e.g., the implications of being in a family characterized by high vs. low discrepancies or high vs. low parental warmth); the current study adds to this by examining within-family associations between perceptions, discrepancies, and parent and adolescent affect (e.g., the implications of having a higher-than-average discrepancy score on a given day). Therefore, this study represents an important adjunct to the current literature about parent–adolescent discrepancies.

Based on the current understanding of perceptions, parent–adolescent discrepancies, and their relationship with adolescent and parent affect, we registered the following hypotheses (https://osf.io/s2u6a):

**Hypothesis 1.** Adolescents’ reports of daily parental warmth will be positively related to adolescents’ reports of daily happiness and negatively to daily sadness and daily irritation.

**Hypothesis 2.** Parents’ reports of daily parental warmth will be positively related to parents’ reports of daily happiness and negatively to daily sadness and daily irritation.

**Hypothesis 3.** Both on the between- and within-family level, adolescent–parent divergence in reports of parental warmth will relate to adolescents’ elevated negative affect and reduced positive affect. Due to lack of information from previous studies, we refrain from making hypothesis about the associations between discrepancies and parents’ affect but will examine the relationship in an exploratory way.

**Hypothesis 4.** Adolescents’ and parents’ daily affect will be positively associated.

We also refrain from stating hypotheses about differences between adolescent–mother and adolescent–father dyads as previous information is inconclusive.

## METHOD

### Method sample I (FLOW)

#### Participants

Participants of sample I consisted of 150 parent–adolescent dyads who participated in the FLOW study, a daily diary study of families in central Pennsylvania, USA. Participants were recruited through local high schools, and data collection occurred from 2014 to 2017. The FLOW study was approved by the Institutional Review Board at Pennsylvania State University (STUDY00000472). Participants completed an eligibility screening to assess their eligibility. In order to be eligible, families had to meet the following criteria: (1) they were a two-caregiver family, (2) the adolescent lived in the house continuously, (3) the family had internet access, (4) participants were fluent in English, (5) the participating adolescent was in 9th or 10th grade, and (6) one parent and one adolescent consented (parent) and or assented (adolescent) to participate. Participating caregivers and adolescents completed baseline surveys before completing a daily diary protocol. Demographics are presented in Table 1. Families’ annual household income ranged from “less than $10,000” to “$125,000 or more,” with a median income between $70,000 and $79,000 per year.

### Procedure

After parents and adolescents consented and assented to participation, they were sent a web-based baseline survey. After completion, a 21-day daily diary protocol was initiated in which the caregiver and adolescent each received a brief (5 min or less) survey via email at 7 p.m. each night for 21 consecutive nights. Participants also received phone call or text message reminders (based on preference) after receiving surveys. Parents and adolescents were compensated separately, earning a $25 Amazon or Wal-Mart gift card (based on preference) for completion of the baseline surveys. For the current study, baseline surveys are only used for measurements of demographic characteristics. For daily surveys, each participant earned $2.50 for the first 4 days of each week, and $5.00 for the last 3 days, for a total of up to $25 per week. Compliance with daily surveys was high, with adolescents completing an average of 19.0 daily surveys (90.4%; SD_Days_ = 2.53) and parents completing an average of 20.3 daily surveys (96.5%; SD_Days_ = 1.28).

### Measures

#### Daily parental warmth

Adolescents were asked one item each day about their perceptions of parental warmth that day: “How warm and affectionate was your [Parent 1] with you.” The text “Parent 1” was replaced with text specific to the participating caregiver for each family (e.g., mother, father, step-mother). Adolescents responded using a digital slider scale from 0 (*not at all true*) to 10 (*very true*), and responses could be adjusted by .10 increments. Parents responded about their own warmth using the parallel item, “I was loving and affectionate with my child.” The parent item used the same response scheme as the adolescent item.

#### Daily affect

Adolescents and parents responded to the same items measuring daily affect. Three facets of daily affect were used in the current study: happiness, sadness, and irritation. Two items assessed each facet. The question stem “How much of the time today did you feel…” was followed by the options “happy” and “content” for happiness, “depressed” and “sad or blue” for sadness, and “angry” and “annoyed” for irritation. Responses ranged from 0 (*none of the time*) to 10 (*all of the time*) and could be adjusted by 0.10 increments. The two items for each construct were averaged for a daily score. Within-person variability (*R*_C_, Bolger & Laurenceau, 2013) and between-person reliability (*R*_*1F*_, Cranford et al., 2006) were calculated. Assessments of happiness, sadness, and irritation showed reliable within-person variability and good between-person reliability across diary days for adolescents (happiness: *R*_*1F*_ = .87, *R*_*C*_ = .80, sadness: *R*_*1F*_ = .86, *R*_*C*_ = .81, and irritation: *R*_*1F*_ = .78, *R*_*C*_ = .72) and parents (happiness: *R*_*1F*_ = .88, *R*_*C*_ = .79, sadness: *R*_*1F*_ = .82, *R*_*C*_ = .82, and irritation: *R*_*1F*_ = .76, *R*_*C*_ = .76).

### Method sample II (RE-PAIR)

#### Participants

Participants of sample II consisted of 80 families who participated in RE-PAIR. RE-PAIR is a Dutch multi–method two-generation study examining the bidirectional interplay between parent–child interactions and adolescent mental well-being by comparing adolescents with a current major depressive disorder or dysthymia and their parents to adolescents without psychopathology and their parents. The complete RE-PAIR study consisted of four parts: online questionnaires, a research day at the lab, 2 weeks of EMA, and a magnetic resonance imaging (MRI)-scan session with the adolescent and one or both parents. Adolescents and one or two parents (if possible) participated. The RE-PAIR study was approved by the Medical Ethics Review Committee (METC) of Leiden University Medical Centre (LUMC; research protocol: P17.241). The current study only used EMA data of the 80 adolescents without psychopathology and their 151 parents. For a detailed description of the in- and exclusion criteria and recruitment of RE-PAIR and this subsample see Janssen, Verkuil, et al., 2021. Demographics are presented in Table 1. Parents indicated monthly family income and reported an income of more than €4.500 (*n* = 79), between €2.500 and €4.500 (*n* = 67), and less than €2.500 (*n* = 5).

### Procedure

All participants signed informed consent. If adolescents were younger than 16 years of age, parents with legal custody also signed informed consent for the adolescent. The Ethica Data application on their own smartphones was used for the EMA, which lasted 14 consecutive days. Participants received four surveys a day (total of 56 questionnaires). The EMA of RE-PAIR in the subsample used in the current study was conducted in the period between September 2018 and November 2019. As compensation for EMA, parents received €20 and adolescents €10. In addition, four gift vouchers of €75 were raffled based on compliance. For detailed information on EMA procedure, time schedule of questionnaires, and compliance see Janssen, Verkuil, et al., 2021.

### Measures

#### Daily parental warmth

Adolescents indicated in the last questionnaire of each day whether they spoke to a parent during that day. If this was the case, they indicated with whom (i.e., mother, father, stepmother, stepfather). Adolescents rated parental warmth for each parent they spoke to by answering the question: “Throughout the day, how warm/loving was your [mother or father] towards you?” Only adolescents’ answers about parents who participated in the EMA were included in the current study. Similarly, parents indicated in the last questionnaire of the day whether they spoke to their adolescent participating in RE-PAIR. Parents rated their own parental warmth by answering the question “Throughout the day, how warm/loving were you toward your child?”. Answers were given on a 7-point Likert-type scale with answer categories ranging from 1 (*not at all*) to 7 (*very*).

#### Daily affect

Adolescents and parents rated their own momentary affect states in every questionnaire (four times a day) with an adapted and shortened version of the Positive and Negative Affect Schedule for Children (PANAS-C; Ebesutani et al., 2012; Watson et al., 1988). In the current study, three affect states were used separately: happiness, irritation, and sadness. These were assessed by asking: “How do you feel at this moment?” followed by: “Happy”, “Sad”, or “Irritated”. Answers were given on a 7-point Likert type scale with answer categories ranging from 1 (*not at all*) to 7 (*very*). A daily mean score of each affect state was calculated. Calculation of within-person variability (*R*_*C*_, Bolger & Laurenceau, 2013) and between-person reliability (*R*_*1F*_, Cranford et al., 2006) was not possible since the affect states were measured with one item. We did calculate the daily average of affect per person per day and correlated this with the momentary affect. The within-person correlations were strong for adolescents (happiness: *r* = .65, *p* < .001: sadness: *r* = .66, *p* < .001, and irritation: *r* = .61, *p* < .001) and parents (happiness: *r* = .61, *p* < .001: sadness: *r* = .62, *p* < .001, and irritation: *r* = .58, *p* < .001).

### Preregistered analysis plan

#### Data preparation

Before performing preregistered (https://osf.io/s2u6a) hybrid models in R Studio version 2022.2.1 (build 461; RStudio Team, 2022) and R version 4.1.3 (R Core Team, 2022), we followed guidelines for centering as presented by Bolger and Laurenceau (2013) and in order to account for the fact that parents’ and adolescents’ ratings of warmth had different ranges, we centered adolescents’ and parents’ scores separately. We subtracted the sample mean from the raw score for centering (a slight deviation from what was presented in the preregistration, in which the raw score was described as being subtracted from the sample mean). To calculate the between-person (or grand mean-centered) score, the sample mean was subtracted from the person mean score. To calculate a within-person centered score, the person mean was subtracted from the daily raw score. Next, we calculated the difference score between adolescent and parent reports of daily parental warmth by subtracting parents’ self-reported warmth from adolescents’ rating of parental warmth on the same day. To calculate a within-person centered score, the dyad mean was subtracted from the daily difference score.

#### Hybrid models

No participants were excluded based on missing data since using compliance thresholds can result in potentially omitting valuable data (Jacobson, 2020). Multilevel hybrid models using maximum likelihood estimation were specified and all available data was used for analysis as this should result in unbiased estimates (Little, 1995). For model building, steps presented by Bolger and Laurenceau (2013) were followed. Three separate hybrid models were estimated using sample I with the different affect states (daily happiness, daily irritation, and daily sadness) as outcomes. The models included fixed effects of adolescent perception of daily parental warmth (between and within-person), parent perception of daily parental warmth (between and within-person), and the difference between adolescent and parent perceptions of daily parental warmth (between and within-person). Day of study was included in the models as predictor. Six separate models were estimated using sample II with the different affect states (daily happiness, daily irritation, and daily sadness) as outcomes, and mother–adolescent and father–adolescent models were run separately. The models included fixed effects of adolescent perception of daily parental warmth (between and within-person), mother or father perception of daily parental warmth (between and within-person), and the difference between adolescent and mother or father perceptions of daily parental warmth (between and within-person). Day of study was included in the models as predictor. For all models, the *p*-values of the unstandardized estimates were interpreted to indicate significance of effects (two-sided, alpha = .05). We do not report effect sizes since there is a lack of consensus on methods to calculate standardized effect sizes in multilevel models (e.g., Wang & Rhemtulla, 2021).

To conceptualize daily parental warmth in sample I we deviated from our preregistration by only using the item that was similar for parents and adolescents (instead of the two items as we proposed). Previous papers using the FLOW data used those two items to conceptualize daily parental warmth. Initially we aimed to be consistent with that, but as the focus of one item differed between parents and adolescents we decided to leave this item out of our main analyses in favor of using only the truly parallel items. Information about the second item as well as correlations, and results of analyses with the two items are presented in Appendix S8. Only small differences were found between the models (with only one difference on the within-person level).

In the result section, we discuss the within-person (daily level) findings. Due to centering of the difference score around each dyad’s average difference score, to capture within-dyad changes, results only provide insight into whether differences are higher or lower than the dyad’s average and not necessarily the absolute directionality of the difference relative to zero (e.g., adolescent reporting higher levels of warmth than parent or parent reporting higher levels of warmth than adolescent). Full information and description of between-person results can be found in the supplementary materials.

## RESULTS

### Results sample I (FLOW)

#### Preliminary analyses

Descriptive statistics of the study variables are shown in Table 2; correlations can be found in Appendix S1. To gain insight into the occurrence of discrepancies between parents’ and adolescents’ reports of parental warmth, we compared their reports at the within-person (i.e., daily) and between-person (i.e., average) level (see Appendix S3 for detailed information on calculation and results at the between-person level). Substantial variation was found regarding discrepancies between adolescent and parent perceptions of daily parental warmth. Based on a cut-off of a discrepancy of more than half SD adolescents and parents had similar perceptions of parental warmth on 58.7% of the days, whereas adolescents reported more parental warmth than parents on 21.2% of days, and on 20.1% of days parents reported more parental warmth than adolescents.

#### Main analyses

**FIGURE 1:**
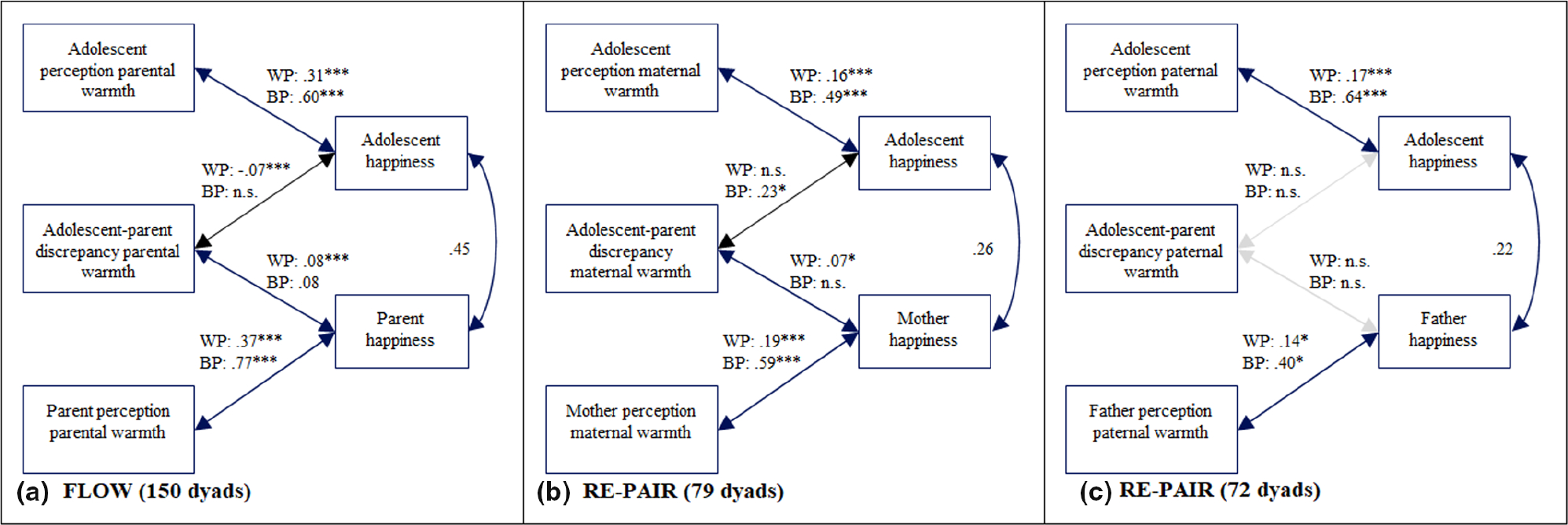
Model results of hybrid models of daily parental warmth and happiness. (a) Results for sample I (*N* = 150 adolescent–parent dyads). (b) Model results for adolescent–mother dyads of the sample II (*n* = 79 dyads). (c) Model results for the adolescent–father dyads of the sample II (*n* = 72 dyads). Unstandardized estimates are shown. W.P., within-person, B.P., between-person. Associations that are not significant on the between- and within-person level are represented by gray lines. *p < .05; ***p < .001.

**FIGURE 2:**
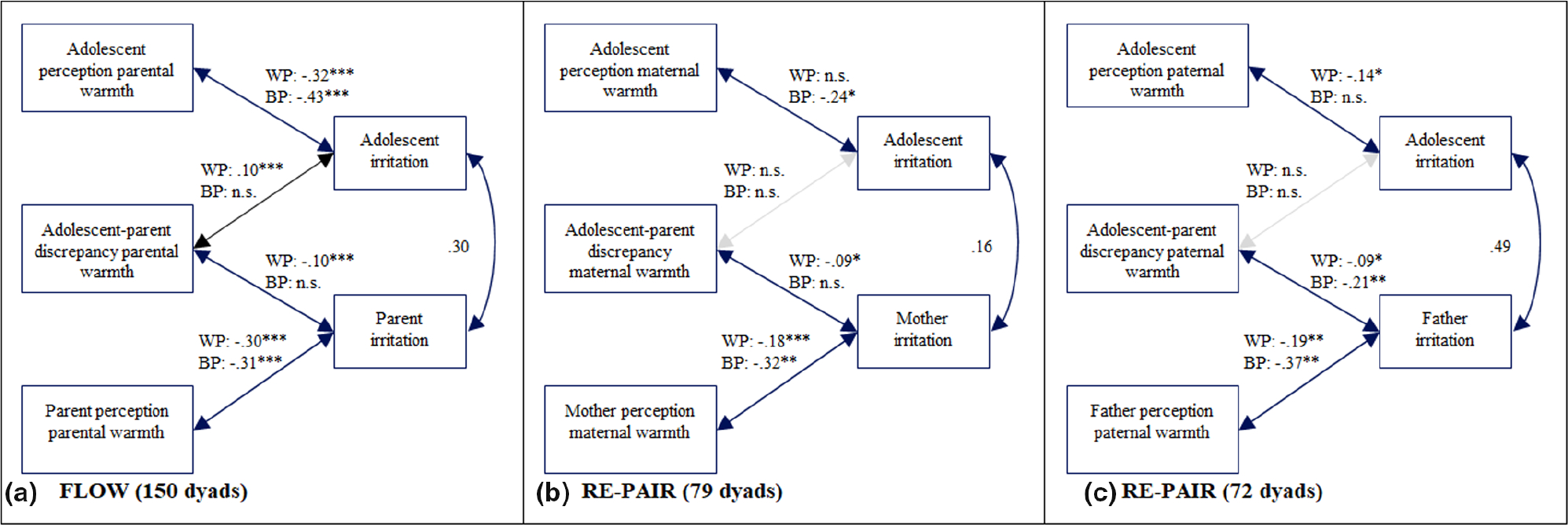
Model results of hybrid models of daily parental warmth and irritation. (a) Results for sample I (*N* = 150 adolescent–parent dyads). (b) Model results for adolescent–mother dyads of the sample II (*n* = 79 dyads). (c) Model results for the adolescent–father dyads of the sample II (*n* = 72 dyads). Unstandardized estimates are shown. W.P., within-person, B.P., between-person. Associations that are not significant on the between- and within-person level are represented by gray lines. *p < .05; **p < .01; ***p < .001.

**FIGURE 3:**
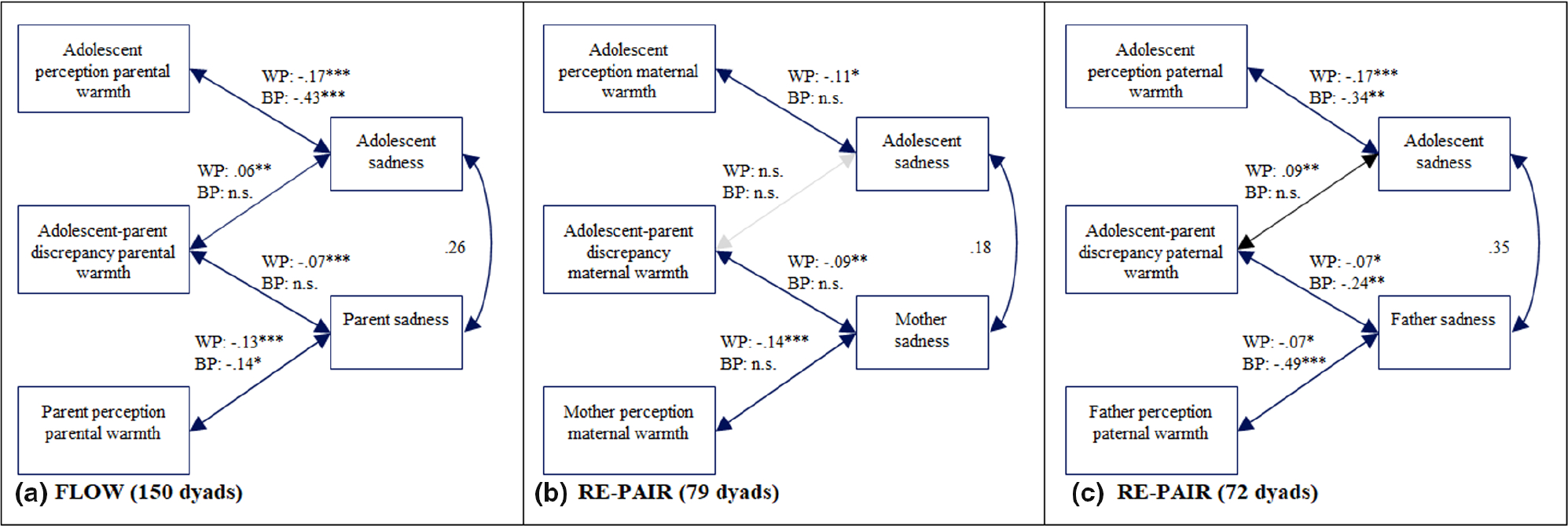
Model results of hybrid models of daily parental warmth and sadness. (a) Results for sample I (*N* = 150 adolescent–parent dyads). (b) Model results for adolescent–mother dyads of the sample II (*n* = 79 dyads). (c) Model results for the adolescent–father dyads of the sample II (*n* = 72 dyads). Unstandardized estimates are shown. W.P., within-person; B.P., between-person. Associations that are not significant on the between- and within-person level are represented by gray lines. *p < .05; **p < .01; ***p < .001.

In order to examine the first aim of the study, whether and how adolescents’ and parents’ perceptions of parental warmth and discrepancies in adolescent–parent reports are related to adolescent and parent affect, three hybrid models were specified. The main results of these models are presented in Figures 1a, 2a, and 3a (see Appendix S4 for full model results). As expected, adolescents’ and parents’ affect were positively associated. In line with the hypotheses, daily fluctuations in parental warmth were related to fluctuations in adolescent affect. On days when adolescents reported higher levels of parental warmth than usual, they reported more happiness (*Est* = .31, *p* < .001), less irritation (*Est* = −.32, *p* < .001), and less sadness (*Est* = −.17, *p* < .001). Similar effects were found for parents. On days when parents reported higher parental warmth than usual, they also reported more happiness (*Est* = .37, *p* < .001), less irritation (*Est* = −0.30, *p* < .001), and less sadness (*Est* = −.13, *p* < .001). Of particular interest was whether the adolescent–parent discrepancies in parental warmth were associated with adolescent and parent affect. On days when this discrepancy was higher than average, adolescents reported less happiness (*Est* = −.07, *p* < .001), more irritation (*Est* = .10, *p* < .001), and more sadness (*Est* = .06, *p* = .003). As expected, the direction of effects was the opposite for parents, consistent with the directional calculation of the difference score (parent report subtracted from adolescent report). On days when the discrepancies were higher than average, parents reported more happiness (*Est* = .08, *p* < .001), less irritation (*Est* = −.10, *p* < .001), and less sadness (*Est* = −.07, *p* < .001).

### Results sample II (RE-PAIR)

#### Preliminary analyses

Descriptive statistics of the study variables are shown in Table 2; correlations can be found in Appendix S1. We compared adolescent–mother and adolescent–father reports of parental warmth at the within-person and between-person level (see Appendix S3 for detailed information on calculation and results at the between-person level). Substantial variation was found regarding discrepancies between adolescent and parent perception of daily parental warmth. Based on a cut-off of a discrepancy of more than half SD adolescents and parents had similar perceptions of parental warmth on 37.0% and 25.2% of the days for mothers and fathers respectively, whereas adolescents reported more daily parental warmth than mothers and fathers respectively on 38.5% and 51.7% of days, and on 24.5% and 23.0% of days mothers and fathers reported more daily parental warmth than adolescents.

#### Main analyses

A total of six hybrid models were specified, in order to examine the first aim and replicate findings from sample I, whether and how adolescents’ and parents’ perceptions of parental warmth and discrepancies in adolescent–parent reports are related to adolescent and parent affect, and the second aim of the study, whether the associations differ between adolescent–mother and adolescent–father dyads.

##### Maternal warmth

Main results of the three models concerning maternal warmth are presented in Figures 1b, 2b, and 3b (see Appendix S5 for full model results and description of between-person results). As expected, adolescents’ and mothers’ affect were positively associated. Most findings regarding the associations between parental warmth and parents’ and adolescents’ affect in the sample I were replicated in sample II. On days when adolescents reported higher levels of maternal warmth than usual, they also reported more happiness (*Est* = .16, *p* = .004) and less sadness (*Est* = −.11, *p* = .037); however, no relations were found with irritation. Regarding mothers’ affect, on days when mothers reported higher parental warmth than usual, they also reported more happiness (*Est* = .19, *p* < .001), less irritation (*Est* = −.18, *p* = .001), and less sadness (*Est* = −0.14, *p* = .001). Regarding mother–adolescent discrepancies in maternal warmth, the discrepancy score was not associated with adolescent daily affect, but was associated with mothers’ affect. On days that the discrepancy between adolescent and mother report of parental warmth was higher than average, mothers reported more happiness (*Est* = .07, *p* = .037), less irritation (*Est* = −.09, *p* = .027), and less sadness (*Est* = −.09, *p* = .002).

##### Paternal warmth

Main results of the three models concerning paternal warmth are presented in Figures 1c, 2c, and 3c (see Appendix S6 for full model results and description of between-person results). As expected, adolescents’ and fathers’ affect were positively associated. Our analyses evaluating paternal warmth also largely replicated findings from sample I. On days when adolescents reported higher levels of paternal warmth than usual, they reported also more happiness (*Est* = .17, *p* = .004), less irritation (*Est* = −.14, *p* = .019), and less sadness (*Est* = −.17, *p* = .001). On days when fathers reported more parental warmth than usual, they also reported more happiness (*Est* = .14, *p* = .010) and less irritation (*Est* = −.19, *p* = .001); however, no relation was found with sadness. Regarding the discrepancies between adolescents and fathers concerning daily paternal warmth, only one association was significantly related to adolescent affect. That is, on days when the discrepancy was higher than average, adolescents reported more sadness (*Est* = .09, *p* = .002), but discrepancies were not associated with happiness or irritation. Discrepancies were more consistently related to fathers’ affect. On days when the discrepancy was higher than average, fathers reported less irritation (*Est* = −.08, *p* = .012) and less sadness (*Est* = −.07, *p* = .015).

### Sensitivity analysis

Interaction terms have been suggested as alternative means of capturing discrepancies in two informants’ reports (De Los Reyes et al., 2013; Laird & de Los Reyes, 2013; Ohannessian et al., 2016). To address concerns regarding the potential redundancy of evaluating difference scores in combination with each of the two informants’ individual scores in a multilevel regression analysis (Laird & de Los Reyes, 2013; Laird & Weems, 2011), we therefore conducted exploratory sensitivity analyses in addition to the preregistered analyses of the hybrid models using only sample I (due to larger sample size). We re-computed our hybrid models using interaction scores to replace discrepancy scores for comparison to our original results. An interaction score was calculated by multiplying parent and adolescent daily reports of parental warmth. Instead of centering on the individual level, we now centered on the dyad level to facilitate interpretation of the interaction score. We centered the interaction score at the within-person level by subtracting the person-mean interaction score from the daily raw score, and at the between-person level by subtracting the sample mean interactions score from the person-mean score (see e.g., Laird & de Los Reyes, 2013 for similar method).

**FIGURE 4:**
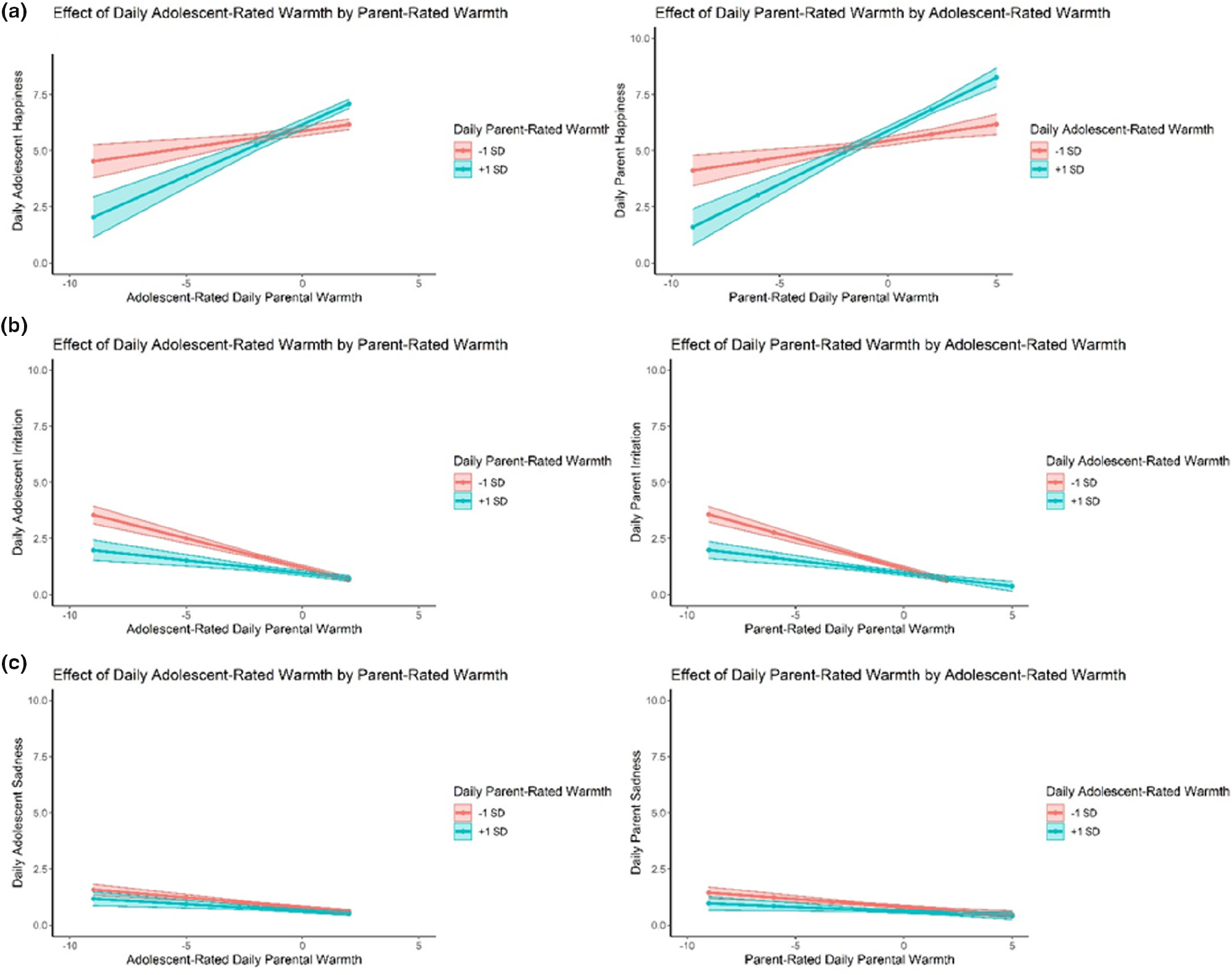
(a–c) Associations between adolescent-rated warmth as a function of parent-rated parental warmth (+1SD vs. −1SD) (left panel) and parent-rated parental warmth as a function of adolescent-rated parental warmth (+1 SD vs. −SD) (right panel) on daily happiness (a), irritation (b), and sadness (c).

Three hybrid models were specified in which adolescent and parent individual daily and average perceptions of warmth, as well as the interactions between adolescent and parent reports of parental warmth, were associated with each of the three parent and adolescent daily affective states (see Table in Appendix S7 for full model results). Regarding parents’ and adolescents’ individual reports of parental warmth, the patterns of results were generally similar to findings using difference scores, reported above. The only difference was in the model including sadness. In the hybrid models including the interaction scores, adolescents’ and parents’ daily sadness was not related to reports of daily parental warmth. Interaction scores between adolescent and parent reports of daily parental warmth were associated with daily adolescent happiness (*Est* = .01, *p* = .007). As shown in Figure 4, adolescent happiness was highest on days when parents and adolescents converged on high reports of parental warmth (e.g., low discrepancy), whereas happiness was lowest on days when adolescents reported low parental warmth and parents reported high parental warmth. Similar findings were found for irritation, with higher levels of irritation when parent and adolescent both report low parental warmth, whereas irritation was lowest on days when parental warmth was rated low by adolescents, regardless of parental ratings (*Est* = −.01, *p* < .001). No interaction was found for sadness (*Est* = −.01, *p* = .051).

Interaction scores of daily parental warmth were associated with parents’ happiness (*Est* = .01, *p* < .001), irritation (*Est* = −.01, *p* < .001), and sadness (*Est* = −.01, *p* < .001) the same day (see Figure 4). For daily happiness of parents, a similar pattern was shown as for adolescent happiness. For daily sadness, parents’ daily sadness was lowest when both parents and adolescents converged on high parental warmth, and parental sadness was highest when parents and adolescents converged on low parental warmth. Parents’ daily irritation was highest when both informants converged on low reports of parental warmth.

It should be noted that the plots in Figure 4 do not fully represent our model results. Due to the required data structure for the hybrid models (e.g., separate rows for each individual), interactions could not be easily probed using traditional methods. Therefore, in order to better understand the interactions, we ran separate models for parent and adolescent outcomes where parent daily and average reports of parental warmth were treated as moderators of adolescent daily and average reports, and vice versa. These were run using the nlme package in R (Pinheiro et al., 2022) and were similar to typical multilevel models with level-one and level-two interactions. We present plots (Figure 4) for each of the interactions at the within-person level, treating parent reports as moderators of adolescent reports for adolescent outcomes models, and treating adolescent reports as moderators of parent reports for parent outcomes.

## DISCUSSION

Since family dynamics stem from perceptions and behaviors of family members that influence each other and interact (Cox & Paley, 1997; Granic et al., 2003; Minuchin, 1985), studies increasingly include both adolescent and parent reports on parenting behavior. Results of these studies showed that these perceptions can differ (Hou et al., 2018, 2020; Korelitz & Garber, 2016) and that the differences between perceptions of parents and adolescents are associated with adolescent general well-being (Hou et al., 2020) as well as daily affect (Janssen, Verkuil, et al., 2021). However, no studies that we know of have taken into account parents’ daily affect, which disregards the interrelatedness of affect between family members. In the current study, we therefore aimed to investigate whether adolescents’ and parents’ perceptions of daily parenting and differences between them relate to daily affect of both adolescents and parents by using novel hybrid models and analyzed parental gender differences.

Our study utilized repeated measures designs, gaining insight into daily fluctuating processes, to assess adolescents and parents across samples of families from two different cultural contexts. Findings were largely consistent with our preregistered hypotheses. Adolescents’ reports of daily parental warmth were positively related to adolescents’ reports of daily happiness and negatively related to daily sadness and irritation. This was the case both on the between-person and within-person level and in both samples supporting our first hypothesis. Similarly, consistent with our second hypothesis, parents’ reports of daily parental warmth were positively related to their own reports of daily happiness and negatively related to daily sadness and irritation. However, with respect to the third hypothesis, testing the relation between discrepancies in reports of parental warmth and adolescent affect, the results were less consistent. For sample I, on days when discrepancies were higher than average (indicating differences between adolescents’ and parents’ reports of parental warmth that were higher than the dyad’s average), adolescents also reported less happiness, more sadness, and more irritation. These findings were not replicated in sample II as divergence in perceptions of maternal warmth was not associated with adolescents’ affect. Adolescents only reported more sadness on days when adolescent–father discrepancies were higher than average. Divergence in adolescent–parent reports of parental warmth was more consistently related to *parents’* affect on the within-person level than to adolescent affect. On days when parents differed more from adolescent report of parental warmth than average, parents reported more happiness, less irritation, and less sadness, which generally was also the case for mothers and fathers in sample II. Lastly, our findings also supported our fourth hypothesis in showing that parents’ and adolescents’ affect were positively associated in the models.

### Adolescent–parent discrepancies and affect in daily life

Most multi-informant studies, including both parents’ and adolescents’ reports of parenting behavior, have supported the idea that divergence in parent–adolescent reports of warmth relate to poorer adolescent well-being in general (e.g., Hou et al., 2020). Although the parent—adolescent relationship can be seen as a dynamic system (Granic et al., 2003) and it has been suggested that divergence in perceptions of the family can undermine both adolescents’ and parents’ well-being (e.g., De Los Reyes, 2011), the relation between parent–adolescent discrepancies and parents’ affect has not yet been evaluated. Our findings in sample I were largely in line with previous studies and suggestions. Generally, discrepancies between adolescents’ and parents’ reports of parental warmth were related to adolescents’ and parents’ affective states. By using repeated measures designs and novel hybrid models (Iida et al., 2018), we were able to gain more insight into the daily dynamic processes of adolescents’ and parents’ perceptions of parenting, discrepancies between them, and its relation to adolescent and parent affect in daily life. Although we did not explicitly focus on differences between the within-person and between-person results, our findings on both levels differ and highlight the importance of examining these dynamic processes on the daily level since individuals’ feelings and perceptions may vary considerably from day- to-day (e.g., Fosco et al., 2019). Although many existing studies have suggested that families who exhibit larger informant discrepancies may be at heightened risk for negative outcomes (e.g., Hou et al., 2020), informant discrepancies may still exhibit meaningful variation in families where discrepancies on average are small. The current study suggests that it may be these day- to-day fluctuations in discrepancies that are most strongly connected to parents’ and adolescents’ daily affect, beyond average level discrepancies.

Our results indicated that divergence between adolescents’ and parents’ reports of parental warmth was more consistently related to parents’ daily affect than adolescents’ daily affect. It could be that discrepancies may reflect parent–adolescent conflict and it is suggested that this impacts parents more strongly than adolescents (e.g., Branje, 2018), but the link between discrepancies, conflict, and adolescent and parent affect has not been tested yet. Another possible explanation is common informant effects (Laird & de Los Reyes, 2013; Laird & Weems, 2011). While both adolescents’ and parents’ reports of parenting behavior are influenced by their own affect, parents report on their own affect and behavior which may therefore be more strongly related than adolescents’ reports. This finding may also suggest that discrepancies in itself do not affect mood, but rather that mood has an impact on the perception of parental behavior. To further elucidate the role of affect on assessing discrepancies in parenting behavior, it is essential for future studies to examine concurrent as well as over time processes. To date, most studies focused on concurrent associations between discrepancies and adolescent well-being and few studies examined the predictive effect of adolescent–parent discrepancies in parenting over time (years; Hou et al., 2020). Results of one of these studies, examining adolescent–parent discrepancies of the parent–adolescent relationships in relation to adolescent depressive symptoms, showed that discrepancies were concurrently linked to more adolescent depressive symptoms but not over time (a year later) when controlling for adolescent depressive symptoms (Nelemans et al., 2016). Understanding how individual affect and other individual factors like mental health and well-being potentially bias reports of parenting is a critical next step in the study of families and family dynamics. This can be especially relevant for clinical contexts, where the bias may potentially be stronger. Given these remaining questions about the directionality between individual affect and informant discrepancies, investigating the bidirectional relation between discrepancies and affect could lead to more insight into the possible influence of affect on perceptions.

Results from our sensitivity analysis, using interaction scores, converged with those from the difference score analysis and strengthen our findings. Moreover, using interaction scores allowed for a more directed and nuanced interpretation of the findings as it provides information on whether high (or low) scores from one informant are relatively more or less strongly associated with the outcome when scores from the other informant are also high (or low) (Laird & de Los Reyes, 2013). For instance, adolescents’ happiness was lowest on days when adolescents reported less warmth than parents. Similarly, for parents, happiness was lowest on days that they reported less parental warmth than adolescents. Convergence on more daily parental warmth was generally related to more happiness, less daily irritation, and sadness for both parents and adolescents. Taken together, our findings highlight the importance of assessing not only perceptions of parenting of adolescent–parent dyads, but also include well-being of both members of the dyad.

### Gender differences for adolescent–mother and adolescent–father dyads

Our findings furthermore indicate differences between adolescent–mother and adolescent–father dyads in the extent to which discrepancies of parental warmth relate to adolescents’ and parents’ affect. Although in sample I, a greater degree of divergence was related to less daily happiness, more daily sadness, and irritation in adolescents, this was not the case in sample II. For adolescent–mother dyads, the discrepancies of parental warmth were not related to adolescent affect and for adolescent–father dyads it was only related to adolescent daily sadness. Differences in design may play a role here. Affect and parenting were both assessed daily in sample I while in sample II parenting was assessed daily but affect was reported four times a day and a mean score was used in the analyses. Moreover, sample I is almost twice the size of sample II, and warmth and affect were assessed for 21 consecutive days instead of 14 days. As the hybrid models are fairly complex, future studies with larger samples are therefore needed.

Additionally, in line with family systems theory (Cox & Paley, 1997; Minuchin, 1985) and conceptualizations of interpersonal emotion dynamics (e.g., Butler, 2011) that emotions within a system can influence each other, we found that adolescents’ and parents’ daily affect were related. This also corresponds with a previous study that showed that daily happiness of adolescents and parents were related (Mercado et al., 2019). However, we did find differences between adolescent–mother and adolescent–father dyads. Happiness of adolescents and mothers was more strongly related compared to adolescents and fathers, while sadness and irritation in adolescent–father dyads were more strongly related compared to adolescent–mother dyads. This supports previous studies showing stronger processes of transmission of affective states between fathers and children (Almeida et al., 1999; Larson & Richards, 1994). It has been suggested that this might have to do with the position of power in the family. Traditionally, fathers often use more power-assertive parenting strategies with children (Youniss & Smollar, 1985); emotions of people with more power may thus impact the family and other family members more. Our findings may also reflect more compartmentalization of affect in mothers compared to fathers (Erel & Burman, 1995; Krishnakumar & Buehler, 2000). Mothers are seen as being more emotion-directed than fathers (De Goede et al., 2009; Mastrotheodoros et al., 2018), and mothers may also be more cautious in showing their irritation or sadness to their children resulting in less transmission of negative affect. This seems to correspond with other findings indicating that fathers are more likely than mothers to spillover tension from the marital dyad to the parent–child dyad (Almeida et al., 1999).

### Limitations and future directions

Even though this study is the first to apply hybrid models to assess the extent to which parent–adolescent discrepancies in daily parental warmth relate to both adolescent’ and parents’ daily affect, several limitations should be acknowledged. The samples, even though from different continents, were fairly homogeneous regarding ethnicity and family constellation. The majority of participants were white and almost all adolescents lived in a two-parent household in both samples. Also, ratings of parental warmth were generally quite high. The consistent findings across the two samples remain a strength of this study. However, findings cannot be generalized to more ethnically diverse samples or families with different family constellations. Moreover, although the current study takes into account fluctuations throughout days, heterogeneity between families was not assessed despite the fact that several previous studies have indicated that these daily life dynamic within-person processes differ between families (e.g., Boele et al., 2020; Janssen, Elzinga, et al., 2021). Future studies should aim to include more racially and socioeconomically diverse samples and assess this heterogeneity to gain more insight.

Furthermore, as the analyses concern concurrent associations, no claims can be made about the direction of the effects. That is, higher discrepancy scores between adolescents’ and parents’ reports of parental warmth could result in less adolescent happiness, but also the other way around, with less happiness yielding higher discrepancies between adolescent–parent perceptions of parental warmth. Research is therefore needed to examine direction of effects. Although the current study separately examined adolescent–mother and adolescent–father dyads, it has been suggested that these dyads are subsystems of a larger system, the family (Restifo & Bögels, 2009). Future studies should aim to include the family as a whole in one model to gain more insight into of the family dynamics. Such studies should include larger samples to ensure sufficient power due to model complexity. Finally, any available method for examining difference scores has limitations. In this case, the centering of difference scores around parent–child dyads’ average scores results in difference scores that reflect a divergence from a dyad’s usual level of discrepancy, but the absolute magnitude of the scores is less interpretable. Using an absolute value discrepancy was also considered, in order to emphasize magnitude of divergence, but in this case information about the direction of divergence would be lost entirely, which was an integral part of our research questions. Emerging methods like the polynomial regression used in our sensitivity analyses may be able to circumvent the issues arising with difference score calculation (Laird & de Los Reyes, 2013).

## CONCLUSION

This study represents the first of its kind to examine parent and adolescent perceptions of daily warmth, as well as discrepancies in reports, in relation to daily parent and adolescent affect. The use of repeated measures in daily life and novel hybrid multilevel models revealed that adolescent–parent discrepancies of parental warmth were more consistently related to parents’ affect than adolescents’ affect in both samples. Our findings imply that the impact of individual affect is more important for perceptions of parenting behavior than the discrepancies between adolescent–parent perceptions for affect. Moreover, differences in interrelatedness of affect between adolescent–mother and adolescent–father dyads support ideas that fathers are less likely to compartmentalize their affect. Future work with larger and more diverse samples should further investigate and unravel the concurrent and over time implications of daily convergence and divergence in parent–adolescent reports in relation to the mood of both adolescent and parents.

## Supplementary Material

Supplementary Material

## Figures and Tables

**TABLE 1:** Sample demographics.

	Flow		Re-Pair	
Variables	*N*		*N*	

Adolescents				
Gender, n Females (%)	150	92 (61.3)	80	51 (63.8)
Age (years), M (SD)	150	14.6 (0.8)	80	15.9 (1.4)
Race/ethnicity % (n)				
White	150	83.3 (125)	80	91.3 (73)
Black/African American		2.7 (4)		1.3 (1)
Asian/Asian American		4.7 (7)		2.5 (2)
Native American/American Indian		0.7 (1)		–
Multiracial		6.7 (10)		3.8 (3)
Hispanic/Latino/a		2.0 (3)		–
Other^a^		0.7 (1)		1.3 (1)
Parents				
Gender, *n* Females (%)	150	143 (95.3)	151	79 (52.3)
Age (years), M (SD)	150	43.4 (6.9)	151	49.0 (5.9)
Race/ethnicity, *n* (%)				
White	150	134 (89.3)	151	147 (97.4)
Black/African American		4 (2.7)		1 (0.7)
Asian/Asian American		5 (3.3)		–
Native American/American Indian		1 (0.7)		–
Multiracial		4 (2.7)		2 (1.3)
Hispanic/Latino/a		2 (1.4)		–
Other^a^		1 (0.7)		1 (0.7)
Relationship with child—mother, *n* (%)				
Biological parent	143	139 (97.2)	79	75 (94.9)
Stepparent		2 (1.4)		–
Foster parent		1 (0.7)		1 (1.3)
Adoptive parent		–		3 (3.8)
Aunt		1 (0.7)		
Relationship with child—father, *n* (%)				
Biological parent	7	7 (100)	72	62(86.1)
Stepparent		–		6 (8.3)
Foster parent		–		3 (4.2)
Adoptive parent		–		1 (1.4)

a Includes Antillean/Surinamese, & Kurdish (RE-PAIR), and West Indian (FLOW). Participants in FLOW could choose multiple values for race/ethnicity, *n* = 2 did not complete information about their own or their child’s race/ethnicity.

**TABLE 2:** Descriptive statistics FLOW and RE-PAIR sample.

			RE-PAIR (sample II)			
	FLOW (sample I)		Adolescent-mother dyads		Adolescent-father dyads	
	M	SD	M	SD	M	SD

Adolescent happiness	8.11	2.26	5.37	1.05	5.36	1.04
Adolescent irritation	1.65	2.23	1.57	0.95	1.58	0.98
Adolescent sadness	1.13	2.17	1.44	0.87	1.45	0.88
Parent happiness	7.61	2.30	5.06	0.93	5.08	0.91
Parent irritation	1.55	2.07	1.61	0.88	1.67	0.91
Parent sadness	0.96	1.80	1.57	0.88	1.62	0.90
Adolescent warmth	8.37	2.31	5.91	1.04	5.80	1.20
Parent warmth	8.14	2.17	5.71	0.94	5.38	0.98

*Note:* For FLOW, parental warmth ranged from 0 (*not at all true*) to 10 (*very true*) and indicators of affect ranged from 0 (*none of the time*) to 10 (*all of the time*). For RE-PAIR, parental warmth and indicators of affect ranged from 1 (*not at all*) to 7 (*very*).General descriptive statistics (not aggregated across days) are provided.
