# Supplementary material for: Adolescents’ and parents’ affect in relation to discrepant perceptions of parental warmth in daily life
Source: J Res Adolesc. Author manuscript; Available in PMC 2024 Sep 26. (PMC11426253; doi:10.1111/jora.12879)
Supplement: Supplementary Material [file NIHMS2021034-supplement-Supplementary_Material.docx]

**Supplementary Materials**

**Adolescents’ and parents’ affect in relation to discrepant perceptions of parental warmth in daily life**

Loes H. C. Janssen^1,2^, Carlie, J. Sloan^3,4^, Bart Verkuil^1,2^, Lisanne A. E. M. Van Houtum^1,2^ Mirjam. C. M. Wever^1,2^, Gregory M. Fosco^3,4^¶, & Bernet M. Elzinga^1,2^¶

^1^Department of Clinical Psychology, Leiden University, Leiden, the Netherlands;

^2^Leiden Institute for Brain and Cognition (LIBC), Leiden, the Netherlands;

^3^Human Development and Family Studies, The Pennsylvania State University, University Park, PA, USA;

^4^Edna Bennett Pierce Prevention Research Center, The Pennsylvania State University, University Park, PA, USA

¶ These authors are Joint Last Authors on this work.

Contact corresponding author l.h.c.janssen@fsw.leidenuniv.nl

**Appendix 1**

Within- and between-person correlations FLOW sample

|  | 1 | 2 | 3 | 4 | 5 | 6 | 7 | 8 |
| --- | --- | --- | --- | --- | --- | --- | --- | --- |
| 1. Adol happiness | – | -0.41*** | -0.47*** | 0.19*** | -0.13*** | -0.10*** | 0.23*** | 0.12*** |
| 2. Adol irritation | -0.59*** | – | 0.46*** | -0.15*** | 0.16*** | 0.13*** | -0.19*** | -.13*** |
| 3. Adol sadness | -0.67*** | 0.83*** | – | -0.12*** | 0.09*** | 0.14*** | -0.14*** | -0.08*** |
| 4. Parent happiness | 0.45*** | -0.31*** | -0.31*** | – | -0.50*** | -0.47*** | 0.14*** | 0.29*** |
| 5. Parent irritation | -0.27*** | 0.36*** | 0.36*** | -0.56*** | – | 0.44*** | -0.13*** | -.20*** |
| 6. Parent sadness | -0.27*** | 0.19** | 0.27*** | -0.52*** | 0.65*** | – | -0.09*** | -.08*** |
| 7. Adol warmth | 0.61*** | -0.37*** | -0.38*** | 0.32*** | -0.15 | -.08 | – | 0.23*** |
| 8. Parent warmth | 0.26** | -0.28*** | -0.26** | 0.67*** | -0.37** | -0.16 | 0.39*** | – |

*Note*: Adol = Adolescent. Between-person and dyad correlations are presented under the diagonal, within-person and dyad correlations are presented above the diagonal.

* *p* < .05; ** *p* < .01; *** *p* < .001

**Appendix 2**

Within- and between-person correlations RE-PAIR sample adolescent-mother dyads

|  | 1 | 2 | 3 | 4 | 5 | 6 | 7 | 8 |
| --- | --- | --- | --- | --- | --- | --- | --- | --- |
| 1. Adol happiness | – | -0.36*** | -0.49*** | 0.08* | -0.09** | -0.06* | 0.11** | 0.00 |
| 2. Adol irritation | -0.54*** | – | 0.35*** | 0.04 | 0.02 | 0.03 | -0.07* | -0.02 |
| 3. Adol sadness | -0.57*** | 0.70*** | – | -0.05 | 0.03 | 0.05 | -0.06 | -0.02 |
| 4. Parent happiness | 0.15 | -0.14 | -0.09 | – | -0.47*** | -0.55*** | 0.11** | 0.12*** |
| 5. Parent irritation | -0.10 | 0.14 | 0.08 | -0.61*** | – | 0.46*** | -0.10** | -0.12*** |
| 6. Parent sadness | -0.13 | 0.15 | 0.12 | -0.63*** | 0.69*** | – | -0.11*** | -0.06 |
| 7. Adol warmth | 0.62*** | -0.32** | 0.38*** | 0.23* | -0.18 | -0.03 | – | 0.15*** |
| 8. Parent warmth | 0.09 | -0.13 | -0.03 | 0.63*** | -0.37*** | -0.22 | 0.42*** | – |

*Note*: Adol = Adolescent. Between-person and dyad correlations are presented under the diagonal, within-person and dyad correlations are presented above the diagonal.

* *p* < .05; ** *p* < .01; *** *p* < .001

Within- and between-person correlations and RE-PAIR sample adolescent-father dyads

|  | 1 | 2 | 3 | 4 | 5 | 6 | 7 | 8 |
| --- | --- | --- | --- | --- | --- | --- | --- | --- |
| 1. Adol happiness | – | -0.36*** | -0.49*** | -0.01 | -0.00 | 0.03 | 0.14*** | 0.09* |
| 2. Adol irritation | -0.54*** | – | 0.35*** | 0.01 | 0.02 | 0.00 | -0.12*** | -0.02 |
| 3. Adol sadness | -0.57*** | 0.70*** | – | 0.07* | -0.06 | -0.07* | -0.18*** | -0.08* |
| 4. Parent happiness | 0.33** | -0.32** | -0.25* | – | -0.47*** | -0.54*** | 0.07 | 0.12** |
| 5. Parent irritation | -0.45*** | 0.51*** | 0.46*** | -0.55*** | – | 0.48*** | -0.10** | -0.14*** |
| 6. Parent sadness | -0.45*** | 0.47*** | 0.42*** | -0.54*** | 0.87*** | – | -0.06 | -0.07 |
| 7. Adol warmth | 0.57*** | -0.25* | -0.31** | 0.15 | -0.22 | -0.25* | – | 0.16*** |
| 8. Parent warmth | 0.23 | -0.14 | -0.23 | 0.33** | -0.23* | -0.27* | 0.22 | – |

*Note*: Adol = Adolescent. Between-person and dyad correlations are presented under the diagonal, within-person and dyad correlations are presented above the diagonal.

* *p* < .05; ** *p* < .01; *** *p* < .001

**Appendix 3**

Occurrence of discrepancies

Adolescents’ and parents’ reports of parenting were compared per day per sample to examine how often adolescent reports were higher than, equal to, and lower than parent reports. A difference more than half a standard deviation between adolescent and parent reports was used as a cut-off (Shanock et al., 2010). To compare on the between-person level an aggregated mean difference score per dyad was calculated to examine how often adolescent reports were higher than, equal to, and lower than parent reports.

*FLOW sample*

Between-person level: 58.7% of the parent-adolescent dyads reported relatively similar perceptions of parental warmth, in 21.2% of the dyads adolescents reported higher parental warmth than parents, and in 20.1% of the dyads parents reported higher parental warmth than adolescents.

*RE-PAIR sample*

Between-person level: 45.6% and 33.3% of adolescents reported relatively similar perceptions as mothers and fathers respectively, in 20.3% and 16.7% dyads adolescents reported lower levels of daily parental warmth than their mothers and fathers, and in 34.2% and 50.0% dyads adolescents reported higher levels of parental warmth than mothers and fathers

**References**

Shanock, L. R., Baran, B. E., Gentry, W. A., Pattison, S. C., & Heggestad, E. D. (2010). Polynomial regression with response surface analysis: a powerful approach for examining moderation and overcoming limitations of difference scores. *Journal of Business and Psychology, 25*, 543–554. doi:10.1007/s10869-010-9183-4**Appendix 4**

Results from hybrid models of relations between daily parental warmth and adolescent and parent affect in FLOW sample

|  | Happy | | Irritation | | Sadness | |
| --- | --- | --- | --- | --- | --- | --- |
| Fixed effects | Est. | (SE) | Est. | (SE) | Est. | (SE) |
| Adolescent |  |  |  |  |  |  |
| Intercept | 7.93*** | 0.12 | 2.12*** | 0.13 | 1.32*** | 0.13 |
| Day | 0.01* | 0.00 | -0.04*** | 0.00 | -0.01** | 0.00 |
| Daily warmth | 0.31*** | 0.04 | -0.32*** | 0.04 | -0.17*** | 0.04 |
| Average warmth | 0.60*** | 0.08 | -0.43*** | 0.08 | -0.43*** | 0.09 |
| Daily diff warmth | -0.07*** | 0.02 | 0.10*** | 0.02 | 0.06** | 0.02 |
| Average diff warmth | -0.02 | 0.07 | 0.15 | 0.08 | 0.13 | 0.08 |
| Parent |  |  |  |  |  |  |
| Intercept | 7.53*** | 0.12 | 1.88*** | 0.12 | 0.99*** | 0.11 |
| Day | 0.01 | 0.00 | -0.03*** | 0.00 | -0.00 | 0.00 |
| Daily warmth | 0.37*** | 0.04 | -0.30*** | 0.03 | -0.13*** | 0.03 |
| Average warmth | 0.77*** | 0.07 | -0.31*** | 0.07 | -0.14* | 0.07 |
| Daily diff warmth | 0.08*** | 0.02 | -0.10*** | 0.02 | -0.07*** | 0.02 |
| Average diff warmth | 0.08 | 0.06 | -0.02 | 0.06 | -0.02 | 0.06 |
| Random effects |  |  |  |  |  |  |
| Adolescent intercept | 1.34 |  | 1.46 |  | 1.47 |  |
| Parent intercept | 1.31 |  | 1.24 |  | 1.19 |  |
| Correlation of between-dyad random effects | 0.45 |  | 0.30 |  | 0.26 |  |
| Adolescent residual | 0.25 |  | 0.32 |  | 0.28 |  |
| Parent residual | 0.26 |  | 0.21 |  | 0.15 |  |
| Correlation of within-dyad residuals | 0.35 |  | -0.04 |  | -0.05 |  |
| Fit indices | AIC  20221 | BIC  20386 | AIC  21263 | BIC  21428 | AIC  20320 | BIC  20485 |

*N* = 150 dyads; Est = unstandardized estimate; SE = Standard Error; AIC = Akaike information criterion; BIC = Bayesian information criterion

* *p* < .05; ** *p* < .01; *** *p* < .001

In line with the expectations, on average, higher adolescent reports of parental warmth were related to more happiness (*Est* = 0.60, *p* < .001), less irritation (*Est* = -0.43, *p* < .001), and less sadness (*Est* = -0.43, *p* < .001). Similar effects were found for parents. In general, parents who reported more parental warmth, also reported more happiness (*Est* = 0.77, *p* < .001), less irritation (*Est* = -0.31, *p* < .001), and less sadness (*Est* = -0.14, *p =* .044). With regard to adolescent-parent difference scores of parental warmth, these were not related to adolescent or parent affect states on average.

**Appendix 5**

Results from hybrid models of relations between daily maternal warmth and adolescent and mother affect RE-PAIR sample

|  | Happy | | Irritation | | Sadness | |
| --- | --- | --- | --- | --- | --- | --- |
| Fixed effects | Est. | (SE) | Est. | (SE) | Est. | (SE) |
| Adolescent |  |  |  |  |  |  |
| Intercept | 5.11*** | 0.09 | 1.54*** | 0.08 | 1.48*** | 0.09 |
| Day | 0.03*** | 0.01 | 0.00 | 0.01 | 0.00 | 0.00 |
| Daily warmth | 0.16** | 0.06 | -0.09 | 0.05 | -0.11* | 0.05 |
| Average warmth | 0.49*** | 0.11 | -0.24* | 0.11 | -0.21 | 0.12 |
| Daily diff warmth | 0.01 | 0.3 | -0.03 | 0.03 | 0.02 | 0.03 |
| Average diff warmth | 0.23* | 0.11 | -0.00 | 0.11 | -0.18 | 0.12 |
| Mother |  |  |  |  |  |  |
| Intercept | 4.94*** | 0.08 | 1.62*** | 0.08 | 1.63*** | 0.08 |
| Day | 0.02** | 0.01 | 0.00 | 0.01 | -0.01* | 0.00 |
| Daily warmth | 0.19*** | 0.05 | -0.18*** | 0.05 | -0.14*** | 0.04 |
| Average warmth | 0.59*** | 0.10 | -0.32** | 0.11 | -0.16 | 0.12 |
| Daily diff warmth | 0.07* | 0.03 | -0.09* | 0.04 | -0.09** | 0.03 |
| Average diff warmth | -0.07 | 0.09 | 0.02 | 0.09 | 0.12 | 0.10 |
| Random effects |  |  |  |  |  |  |
| Adolescent intercept | 0.60 |  | 0.57 |  | 0.67 |  |
| Mother intercept | 0.52 |  | 0.52 |  | 0.62 |  |
| Correlation of between-dyad random effects | 0.26 |  | 0.16 |  | 0.18 |  |
| Adolescent residual | 0.23 |  | 0.20 |  | 0.26 |  |
| Mother residual | 0.08 |  | 0.06 |  | 0.09 |  |
| Correlation of within-dyad residuals | 0.06 |  | 0.66 |  | 0.88 |  |
| Fit indices | AIC  3157 | BIC  3290 | AIC  3196 | BIC  3323 | AIC  2670 | BIC  2803 |

*N* = 79 dyads; Est = unstandardized estimate; SE = Standard Error; AIC = Akaike information criterion; BIC = Bayesian information criterion

* *p* < .05; ** *p* < .01; *** *p* < .001

On average, higher adolescent reports of maternal warmth were related to more happiness (*Est* = 0.49, *p* < .001) and less irritation *(Est* = -0.24, *p* = .027), but not to sadness of adolescents. Mothers who reported more warmth, also reported more happiness (*Est* = 0.59, *p* < .001) and less irritation (*Est* = -0.32, *p* = .003) in general, but warmth was not related to sadness. With regard the mother-adolescent discrepancies in maternal warmth, it was found that the average difference score was only significantly related to adolescent happiness (*Est* = 0.23, *p* = .044). Dyads characterized by higher discrepancies tended to have adolescents reporting more happiness. Between-person differences in average discrepancy magnitude were not related to affective states of mothers.

**Appendix 6**

Results from hybrid models of relations between daily paternal warmth and adolescent and father affect RE-PAIR sample

|  | Happy | | Irritation | | Sadness | |
| --- | --- | --- | --- | --- | --- | --- |
| Fixed effects | Est. | (SE) | Est. | (SE) | Est. | (SE) |
| Adolescent |  |  |  |  |  |  |
| Intercept | 5.16*** | 0.10 | 1.62*** | 0.10 | 1.49*** | 0.09 |
| Day | 0.03*** | 0.01 | -0.01 | 0.01 | -0.00 | 0.00 |
| Daily warmth | 0.17** | 0.06 | -0.14* | 0.06 | -0.17*** | 0.05 |
| Average warmth | 0.64*** | 0.12 | -0.17 | 0.12 | -0.34** | 0.11 |
| Daily diff warmth | -0.06 | 0.04 | 0.07 | 0.04 | 0.09** | 0.03 |
| Average diff warmth | -0.16 | 0.10 | -0.02 | 0.10 | 0.17 | 0.10 |
| Father |  |  |  |  |  |  |
| Intercept | 5.01*** | 0.09 | 1.74*** | 0.10 | 1.69*** | 0.09 |
| Day | 0.01 | 0.01 | -0.01 | 0.01 | -0.01 | 0.01 |
| Daily warmth | 0.14* | 0.05 | -0.19** | 0.06 | -0.09 | 0.05 |
| Average warmth | 0.40** | 0.12 | -0.37** | 0.12 | -0.49*** | 0.12 |
| Daily diff warmth | 0.06 | 0.03 | 0.08* | 0.03 | -0.07* | 0.03 |
| Average diff warmth | 0.09 | 0.08 | -0.21** | 0.08 | -0.24** | 0.08 |
| Random effects |  |  |  |  |  |  |
| Adolescent intercept | 0.65 |  | 0.68 |  | 0.65 |  |
| Father intercept | 0.65 |  | 0.67 |  | 0.65 |  |
| Correlation of between-dyad random effects | 0.22 |  | 0.49 |  | 0.35 |  |
| Adolescent residual | 0.22 |  | 0.27 |  | 0.24 |  |
| Father residual | 0.20 |  | 0.28 |  | 0.20 |  |
| Correlation of within-dyad residuals | -0.05 |  | -0.13 |  | 0.78 |  |
| Fit indices | AIC  2379 | BIC  2506 | AIC  2320 | BIC  2447 | AIC  1955 | BIC  2083 |

*N* = 72 dyads; Est = unstandardized estimate; SE = Standard Error; AIC = Akaike information criterion; BIC = Bayesian information criterion

* *p* < .05; ** *p* < .01; *** *p* < .001

Generally, adolescents reports of more paternal warmth were related to more happiness (*Est* = 0.64, *p* < .001), and less sadness (*Est* = -0.34, *p* = .003), but not to irritation. For fathers, more reported parental warmth was related to more happiness (*Est* = 0.40, *p* = .001), less irritation (*Est* = -0.37, *p* = .003), and less sadness (*Est* = -0.49, *p* < .001) on average. At the between-person level, differences in average father-adolescent discrepancies were associated with less irritation (*Est* = -0.21, *p* = .008) and less sadness (*Est* = -0.24, *p* = .003). Dyads characterized by higher discrepancies tended to have fathers who reported less irritation and sadness.

**Appendix 7**

Results from hybrid models of relations between daily parental warmth interactions and parent and adolescent daily affect

|  | Happy | | Irritation | | Sadness | |
| --- | --- | --- | --- | --- | --- | --- |
| Fixed effects | Est. | (SE) | Est. | (SE) | Est. | (SE) |
| Adolescent |  |  |  |  |  |  |
| Intercept | 7.92*** | 0.12 | 2.14*** | 0.13 | 1.32*** | 0.13 |
| Day | 0.01* | 0.00 | -0.03*** | 0.00 | -0.01** | 0.00 |
| Daily warmth | 0.19*** | 0.04 | -0.19*** | 0.05 | -0.07 | 0.05 |
| Average warmth | 0.52*** | 0.10 | -0.20 | 0.11 | -0.19 | 0.12 |
| Daily Parent x Adol. Int. | 0.01** | 0.00 | -0.01*** | 0.00 | -0.01 | 0.00 |
| Average Parent x Adol. Int. | 0.01 | 0.01 | -0.01 | 0.01 | -0.01 | 0.01 |
| Parent |  |  |  |  |  |  |
| Intercept | 7.53*** | 0.12 | 1.88*** | 0.12 | 0.99*** | 0.11 |
| Day | 0.01 | 0.00 | -0.03*** | 0.00 | -0.00 | 0.00 |
| Daily warmth | 0.21*** | 0.04 | -0.11* | 0.05 | 0.02 | 0.04 |
| Average warmth | 0.62*** | 0.11 | -0.18 | 0.11 | -0.08 | 0.10 |
| Daily Parent x Adol. Int. | 0.01** | 0.00 | -0.01*** | 0.00 | -0.01** | 0.00 |
| Average Parent x Adol. Int. | 0.01 | 0.01 | -0.01 | 0.00 | -0.00 | 0.01 |
| Random effects |  |  |  |  |  |  |
| Adolescent intercept | 1.34 |  | 1.47 |  | 1.48 |  |
| Parent intercept | 1.30 |  | 1.24 |  | 1.19 |  |
| Correlation of between-dyad random effects | 0.45 |  | 0.30 |  | 0.26 |  |
| Adolescent residual | 0.25 |  | 0.32 |  | 0.27 |  |
| Parent residual | 0.26 |  | 0.21 |  | 0.15 |  |
| Correlation of within-dyad residuals | 0.34 |  | 0.03 |  | -0.04 |  |
| Fit indices | AIC  20227 | BIC  20393 | AIC  21273 | BIC  21438 | AIC  20325 | BIC  20490 |
| *N* = 150 dyads; Est = unstandardized estimate; SE = Standard Error; AIC = Akaike information criterion; BIC = Bayesian information criterion; Adol. = Adolescent; Int. = Interaction Score  * *p* < .05; ** *p* < .01; *** *p* < .001 | | | | | | |

At the between-person level, average interaction scores were not associated with differences in adolescent happiness, irritation, or sadness. With regard to parents, average interaction scores were also not associated with differences in parent happiness, irritation, or sadness.

**Appendix 8**

Descriptive statistics and results of analyses with daily parental warmth measure containing 2 items FLOW

Adolescents were asked two items each day about their perceptions of parental warmth that day, “How warm and affectionate was your [Parent 1] with you,” and “ How much did your [Parent 1] care about your feelings?” The text “Parent 1” in each question was replaced with text specific to the participating caregiver for each family (e.g., mother, father, step-mother). Adolescents responded using a digital slider scale from 0 (Not at All True) to 10 (Very True), and responses could be adjusted by .10 increments. Parents responded about their own warmth using the parallel items, “I was loving and affectionate with my child,” and “I tried to understand my child’s point of view. ” Parent items used the same response scheme as adolescent items.

Within- and between-person correlations FLOW sample

|  | 1 | 2 | 3 | 4 | 5 | 6 | 7 | 8 |
| --- | --- | --- | --- | --- | --- | --- | --- | --- |
| 1. Adol happiness | – | -0.41*** | -0.47*** | 0.19*** | -0.13*** | -0.10*** | 0.25*** | 0.11*** |
| 2. Adol irritation | -0.59*** | – | 0.46*** | -0.15*** | 0.16*** | 0.13*** | -0.20*** | -.13*** |
| 3. Adol sadness | -0.67*** | 0.83*** | – | -0.12*** | 0.09*** | 0.14*** | -0.17*** | -0.05** |
| 4. Parent happiness | 0.45*** | -0.31*** | -0.31*** | – | -0.50*** | -0.47*** | 0.16*** | 0.28*** |
| 5. Parent irritation | -0.27*** | 0.36*** | 0.36*** | -0.56*** | – | 0.44*** | -0.15*** | -.18*** |
| 6. Parent sadness | -0.27*** | 0.19** | 0.27*** | -0.52*** | 0.65*** | – | -0.11*** | -.09*** |
| 7. Adol warmth | 0.61*** | -0.36*** | -0.39*** | 0.34*** | -0.20* | -.13 | – | 0.21*** |
| 8. Parent warmth | 0.25** | -0.24*** | -0.20** | 0.61*** | -0.31* | -0.11 | 0.32*** | – |

*Note*: Adol = Adolescent. Between-person and dyad correlations are presented under the diagonal, within-person and dyad correlations are presented above the diagonal.

* *p* < .05; ** *p* < .01; *** *p* < .001

Occurrence of discrepancies

*FLOW sample*

Between-person level: 58.7% of the parent-adolescent dyads reported relatively similar perceptions of parental warmth, in 21.3% of the dyads adolescents reported higher parental warmth than parents, and in 20% of the dyads parents reported higher parental warmth than adolescents.

Results from hybrid models of relations between daily parental warmth and adolescent and parent affect in FLOW sample

|  | Happy | | Irritation | | Sadness | |
| --- | --- | --- | --- | --- | --- | --- |
| Fixed effects | Est. | (SE) | Est. | (SE) | Est. | (SE) |
| Adolescent |  |  |  |  |  |  |
| Intercept | 7.91*** | 0.12 | 2.13*** | 0.13 | 1.32*** | 0.13 |
| Day | 0.01** | 0.00 | -0.04*** | 0.00 | -0.01** | 0.00 |
| Daily warmth | 0.36*** | 0.04 | -0.39*** | 0.05 | -0.19*** | 0.04 |
| Average warmth | 0.67*** | 0.08 | -0.44*** | 0.09 | -0.44*** | 0.09 |
| Daily diff warmth | -0.07** | 0.02 | 0.10*** | 0.03 | 0.02 | 0.02 |
| Average diff warmth | -0.08 | 0.07 | 0.14 | 0.08 | 0.10 | 0.08 |
| Parent |  |  |  |  |  |  |
| Intercept | 7.53*** | 0.12 | 1.88*** | 0.12 | 0.99*** | 0.11 |
| Day | 0.01 | 0.00 | -0.03*** | 0.00 | -0.00 | 0.00 |
| Daily warmth | 0.43*** | 0.04 | -0.36*** | 0.04 | -0.19*** | 0.03 |
| Average warmth | 0.79*** | 0.08 | -0.32*** | 0.08 | -0.13 | 0.07 |
| Daily diff warmth | 0.11*** | 0.02 | -0.14*** | 0.02 | -0.10*** | 0.02 |
| Average diff warmth | 0.15* | 0.07 | -0.07 | 0.06 | -0.06 | 0.06 |
| Random effects |  |  |  |  |  |  |
| Adolescent intercept | 1.33 |  | 1.47 |  | 1.48 |  |
| Parent intercept | 1.38 |  | 1.27 |  | 1.20 |  |
| Correlation of between-dyad random effects | 0.37 |  | 0.30 |  | 0.26 |  |
| Adolescent residual | 0.26 |  | 0.35 |  | 0.27 |  |
| Parent residual | 0.28 |  | 0.27 |  | 0.20 |  |
| Correlation of within-dyad residuals | 0.54 |  | 0.12 |  | 0.35 |  |
| Fit indices | AIC  20309 | BIC  20475 | AIC  21354 | BIC  21520 | AIC  20383 | BIC  20548 |

*N* = 150 dyads; Est = unstandardized estimate; SE = Standard Error; AIC = Akaike information criterion; BIC = Bayesian information criterion

* *p* < .05; ** *p* < .01; *** *p* < .001

In line with the expectations, on average, higher adolescent reports of parental warmth were related to more happiness (*Est* = 0.67, *p* < .001), less irritation (*Est* = -0.44, *p* < .001), and less sadness (*Est* = -0.44, *p* < .001). Similar effects were found for parents. In general, parents who reported more parental warmth, also reported more happiness (*Est* = 0.79, *p* < .001), less irritation (*Est* = -0.32, *p* < .001), and less sadness (*Est* = -0.13, *p =* .062). With regard to adolescent-parent difference scores of parental warmth, it was not related to adolescent affect states on average. For parents, differences in average parent-adolescent discrepancies were only associated with parents’ happiness (*Est* = 0.15, *p* = .024), with dyads characterized by higher discrepancies tending to have parents who reported more daily happiness.

Results from hybrid models of relations between daily parental warmth interactions and parent and adolescent daily affect

|  | Happy | | Irritation | | Sadness | |
| --- | --- | --- | --- | --- | --- | --- |
| Fixed effects | Est. | (SE) | Est. | (SE) | Est. | (SE) |
| Adolescent |  |  |  |  |  |  |
| Intercept | 7.91*** | 0.12 | 2.14*** | 0.13 | 1.32*** | 0.13 |
| Day | 0.01** | 0.00 | -0.03*** | 0.00 | -0.01* | 0.01 |
| Daily warmth | 0.25*** | 0.04 | -0.19*** | 0.05 | -0.15** | 0.05 |
| Average warmth | 0.47*** | 0.10 | -0.20 | 0.11 | -0.26* | 0.11 |
| Daily Parent x Adol. Int. | 0.01* | 0.00 | -0.01*** | 0.00 | -0.00 | 0.00 |
| Average Parent x Adol. Int. | 0.01 | 0.01 | -0.01 | 0.01 | -0.01 | 0.01 |
| Parent |  |  |  |  |  |  |
| Intercept | 7.53*** | 0.12 | 1.88*** | 0.12 | 0.99*** | 0.11 |
| Day | 0.01 | 0.00 | -0.03*** | 0.00 | -0.00 | 0.00 |
| Daily warmth | 0.21*** | 0.04 | -0.11* | 0.05 | 0.01 | 0.04 |
| Average warmth | 0.49*** | 0.12 | -0.18 | 0.11 | 0.01 | 0.10 |
| Daily Parent x Adol. Int. | 0.01*** | 0.00 | -0.01*** | 0.00 | -0.01*** | 0.00 |
| Average Parent x Adol. Int. | 0.02* | 0.01 | -0.01 | 0.00 | -0.01 | 0.01 |
| Random effects |  |  |  |  |  |  |
| Adolescent intercept | 1.32 |  | 1.47 |  | 1.48 |  |
| Parent intercept | 1.38 |  | 1.27 |  | 1.19 |  |
| Correlation of between-dyad random effects | 0.36 |  | 0.30 |  | 0.26 |  |
| Adolescent residual | 0.26 |  | 0.35 |  | 0.27 |  |
| Parent residual | 0.28 |  | 0.28 |  | 0.21 |  |
| Correlation of within-dyad residuals | 0.52 |  | 0.11 |  | 0.36 |  |
| Fit indices | AIC  20313 | BIC  20479 | AIC  21365 | BIC  21531 | AIC  20385 | BIC  20551 |
| *N* = 150 dyads; Est = unstandardized estimate; SE = Standard Error; AIC = Akaike information criterion; BIC = Bayesian information criterion; Adol. = Adolescent; Int. = Interaction Score  * *p* < .05; ** *p* < .01; *** *p* < .001 | | | | | | |

At the between-person level, average interaction scores were not associated with differences in adolescent happiness, irritation, or sadness. With regard to parents, average interaction scores were related to parental happiness (*Est* = 0.02, p = .039).
